# SURFXsim: Free-Energy Simulations of Spin-Forbidden Events with a Spin-Gap Collective Variable

**DOI:** 10.1021/acs.jctc.6c00796

**Published:** 2026-07-25

**Authors:** Ajmal Rahman Mullukkandy, Bernd Ensing, Carles Bo, Albert Solé-Daura

**Affiliations:** † Department of Physical and Inorganic Chemistry, 16777 Universitat Rovira i Virgili, Marcel lí Domingo, 1, 43007 Tarragona, Spain; ‡ Institute of Chemical Research of Catalonia (ICIQ-CERCA), 202569 The Barcelona Institute of Science and Technology, Avgda. Països Catalans, 16, 43007 Tarragona, Spain; § Van ’t Hoff Institute for Molecular Sciences and Amsterdam Center for Multiscale Modeling, University of Amsterdam, Science Park 904, 1098 XH Amsterdam, The Netherlands

## Abstract

Spin-forbidden processes are central to catalysis, photochemistry, and transition-metal reactivity, yet their simulation remains challenging because the relevant transformations proceed between potential energy surfaces of different spin multiplicities and are frequently thermally activated rare events. Existing computational strategies typically provide either static descriptions of crossing regions, such as minimum-energy crossing point searches, or dynamically explicit but computationally demanding nonadiabatic simulations that are difficult to apply to medium- and large-sized systems. Here, we present SURFXsim, a computational framework for simulating spin-forbidden events by combining atomistic molecular dynamics, a configuration-dependent mixed Hamiltonian, and enhanced sampling along a spin-gap collective variable. The method should be understood as a finite-temperature sampling and interpolation strategy, rather than as a direct simulation of coherent spin–orbit-coupled electronic-state dynamics. It enables smooth propagation between end-state basins while using metadynamics to drive the exploration of crossing regions and reconstruct free-energy landscapes. SURFXsim is implemented in Python on top of ASE and interfaced with PLUMED, allowing the use of both machine-learning and conventional electronic-structure calculators. We illustrate the approach with distortion-driven and reactivity-driven spin-crossing examples using machine learning potentials and further demonstrate compatibility with a quantum-mechanical backend through the Gaussian package. Together, these results position SURFXsim as a practical and flexible route for studying spin-forbidden chemistry beyond the scale routinely accessible to fully nonadiabatic dynamics.

## Graphical abstract

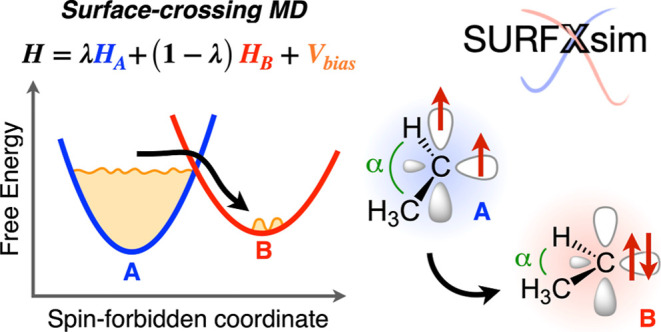

## Introduction

Spin-forbidden reactions, in which chemical transformations proceed between states of different spin multiplicity, are fundamental to a broad range of problems in chemistry. Such processes are central to homogeneous and heterogeneous catalysis, photochemistry, transition-metal reactivity, and functional materials, where changes in spin state can determine both reactivity and selectivity.^1–7^ Although spin-selection rules formally disfavor these events, spin changes can still occur efficiently when thermal fluctuations or structural rearrangements bring the system into regions where two potential energy surfaces of different spin multiplicities become energetically competitive. Typically, the thermally activated population of these regions slightly mixes the spin states through spin–orbit coupling, lifting the strict forbiddenness. Describing how molecular systems approach, cross, and leave these regions is therefore essential for a mechanistic understanding of many chemically relevant transformations.

Computationally, spin-forbidden events are notoriously challenging. Static quantum-chemical approaches have long been used to characterize crossing regions, often by exploring selected internal coordinates or by locating minimum-energy crossing points (MECPs) between two potential energy surfaces.^8–14^ MECP optimizations became broadly accessible to the community through the conjugate-gradient algorithm introduced by Harvey.^15^ As a result, they have been especially widely adopted because they provide direct access to the lowest-energy point at which two states intersect, and when combined with density functional theory, they can be applied to chemically meaningful systems at moderate cost. Yet static approaches provide only a localized view of the crossing region and do not directly reveal the finite-temperature dynamics, entropic effects, or free-energy barriers that govern whether a spin change will occur under realistic conditions.

At the opposite end of the spectrum, nonadiabatic molecular dynamics and related trajectory-based methods can provide a more explicit dynamical picture of spin-forbidden chemistry.^16–21^ However, these approaches are still computationally demanding, particularly when the underlying electronic structure must be described with multiconfigurational wave function methods or repeated excited-state calculations. As a result, practical applications are often limited to small systems, short time scales, or barrierless processes that occur downhill on the potential energy surface or involve overcoming very small barriers. This leaves a significant methodological gap for medium-to large-sized systems in which density functional theory or machine-learning potentials are the only affordable atomistic models and where the chemically relevant event is a thermally activated crossing that requires enhanced sampling.

Recent efforts have begun to address part of this problem. In particular, enhanced-sampling-based strategies that bias descriptors of the crossing region, such as the off-diagonal terms of the overlap matrix between both states, offer a promising route to driving rare spin-changing events and mapping their associated free energy landscapes.^22^ While these developments are highly valuable and complementary to more rigorous nonadiabatic treatments, the computational cost associated with repeated high-level electronic-structure evaluations remains a major bottleneck for realistic systems, especially when long trajectories or extensive sampling are required. More broadly, there remains a need for a practical atomistic framework that can combine dynamic sampling of spin crossings with flexible, affordable back-end calculators.

Here, we introduce SURFXsim, a computational framework designed to fill this niche. SURFXsim combines three key ingredients: (i) a configuration-dependent mixed Hamiltonian that smoothly interpolates between two spin-state end points, (ii) a novel spin-gap collective variable that naturally describes the reaction coordinate and identifies the crossing region, and (iii) enhanced sampling, here implemented through metadynamics,^23^ to drive and analyze spin-forbidden transitions. In this sense, SURFXsim is closer in spirit to alchemical or λ-dynamics sampling methods than to explicit nonadiabatic dynamics: intermediate mixed-Hamiltonian configurations are used to connect the two physical end-state ensembles and to identify thermally accessible crossing geometries. The method is implemented in Python on top of ASE^24^ and coupled to PLUMED,^25^ making it straightforward to interface with both machine-learning and quantum-chemistry backends. In this work, we demonstrate the method for two representative classes of spin-forbidden processes using the UMA^26^ MLP for the sake of computational efficiency: a distortion-driven spin crossover in a [Fe­(2-pic)_3_]^2+^ (2-pic = 2-picolylamine) model system and a reactivity-driven spin crossover in a carbonyl iron complex for which the spin-gap CV is combined with a bond-distance coordinate in two-dimensional metadynamics. We further show that the same strategy is compatible with DFT potentials, exemplified by a Gaussian^27^-based simulation for methylcarbene. Together, these examples illustrate that SURFXsim provides a practical route to simulating spin-forbidden events as finite-temperature rare processes on multidimensional free-energy landscapes. Note that since the main objective of this work is methodological proof of concept, the emphasis is on demonstrating the robustness and flexibility of the SURFXsim framework rather than on quantitatively benchmarking every backend potential for every test system.

## Methodology

### Conceptual Framework

SURFXsim stands for “SURFace CROSSing simulation” and is designed to simulate spin-forbidden processes within an atomistic molecular dynamics framework while avoiding the cost of fully nonadiabatic dynamics. The core idea is to propagate the nuclei on a mixed Hamiltonian constructed from two pure electronic states of different spin multiplicity and to bias the trajectory along a collective variable that directly measures the instantaneous spin gap. This combination allows the system to evolve smoothly between two spin states and enables the use of enhanced-sampling methods to explore rare spin-changing events.

The implementation was intentionally modular. At every simulation step, the code queries an external calculator for the energies and forces of two states, denoted as *A* and *B*. These may correspond, for example, to singlet and triplet or singlet and quintet states depending on the problem of interest. From these quantities, SURFXsim builds a mixed Hamiltonian, computes the spin-gap collective variable, and communicates the relevant energies and forces to PLUMED, which applies the bias potential and returns the bias forces needed for molecular dynamics propagation. The workflow of the code is illustrated in Figure 1.

**Figure 1:**
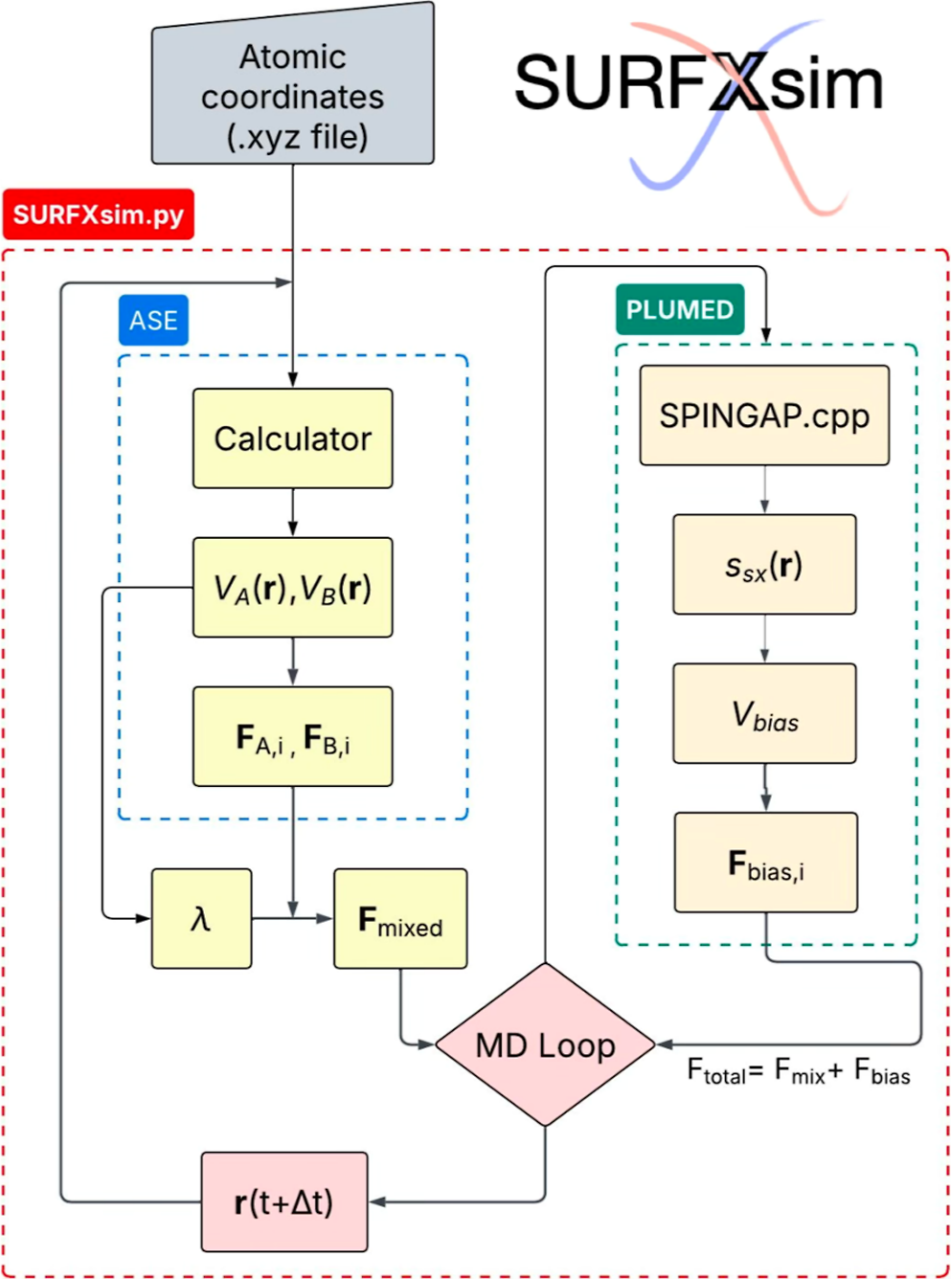
Overview of the SURFXsim computational workflow, illustrating its main components involved in the MD simulation loop.

### Mixed Hamiltonian

To allow a molecular dynamics trajectory to move continuously between two spin states, we propagate the nuclei on a mixed Hamiltonian

1 $$H_{mixed}\left(r,p\right)=\lambda H_{A}\left(r,p\right)+\left(1-\lambda \right)H_{B}\left(r,p\right)$$

In eq 1, *r* = (*r*_1_, ..., *r*_*N*_) and *p* = (*p*_1_, ..., *p*_*N*_) are the nuclear coordinates and momenta, respectively, and λ controls the relative contribution of the two states. In the present implementation, λ depends on the instantaneous energy difference

2 $$\Delta V\left(r\right)=V_{B}\left(r\right)-V_{A}\left(r\right)$$

defined in eq 2, through the Boltzmann-like expression in eq 3

3 $$\lambda \left(\Delta V\right)=\frac{1}{1+\exp\left(\Delta V/k_{B}T\right)}$$

This choice has several desirable properties. As shown in Figure 2, when state *A* is much lower in energy than state *B*, λ approaches unity and the dynamics is effectively propagated on state *A*. Conversely, when state *B* is more stable, λ approaches zero. At the crossing region, where the two states are degenerate, λ = 0.5, so both states contribute equally to the mixed Hamiltonian. The parameter λ is therefore an energy-gap-dependent interpolation coordinate used by the sampling algorithm. It should not be interpreted as a spin–orbit-coupling mixing angle, as a quantum amplitude, or as a Boltzmann population of two coherently coupled electronic states. In a real molecule, spin–orbit coupling would generate adiabatic mixed states whose composition depends on the coupling matrix element and the local energy gap. The present mixed Hamiltonian instead provides a continuous classical potential on which the nuclei can be propagated without force discontinuities while the trajectory is guided toward configurations where the two diabatic spin-state energies are similar. This leads to a smooth interpolation of energies and forces

4 $$V_{mixed}=\lambda V_{A}+\left(1-\lambda \right)V_{B}$$

5 $$F_{mixed,i}=\lambda F_{A,i}+\left(1-\lambda \right)F_{B,i}$$

as given in eqs 4 and 5, respectively, which prevents abrupt force discontinuities when the system approaches the crossing region.

**Figure 2:**
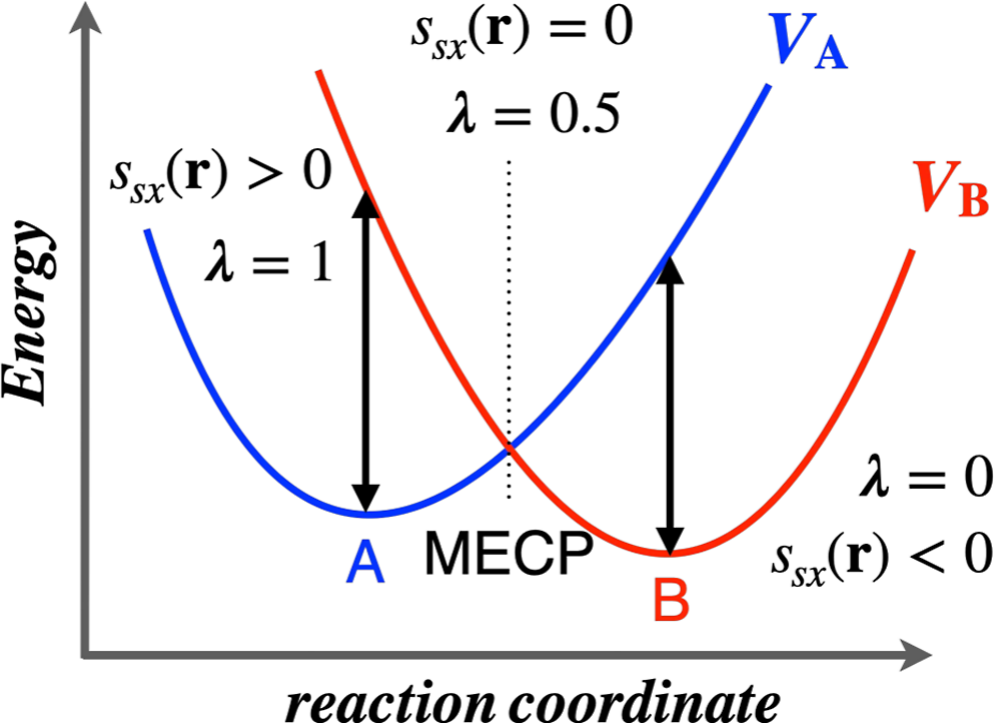
Schematic representation of a reaction coordinate involving a minimum-energy crossing point (MECP) between two potential energy surfaces corresponding to two pure electronic states (A,B) (blue and red curves, respectively) of distinct spin multiplicity. Values of the collective variable *s*_sx_(*r*) and the mixing parameter λ are shown at three key regions of the reaction coordinate.

The mixed-Hamiltonian treatment used here should be viewed as a practical interpolation strategy that allows atomistic sampling of spin-crossing pathways at finite temperatures. It does not aim to reproduce the full quantum mechanical details of nonadiabatic dynamics. Rather, its purpose is to provide a chemically meaningful and computationally affordable route for exploring the structural and free energy consequences of approaching spin-state crossings in systems that would otherwise be inaccessible. Just as it is impractical to simulate protein dynamics directly with DFT because of the prohibitive computational cost, we adopt a more pragmatic approach to investigate the mechanisms of spin-forbidden pathways. Accordingly, free energy profiles reconstructed along *s*_sx_ should be interpreted as landscapes associated with this mixed-Hamiltonian sampling protocol and with the two underlying diabatic spin-state energies. The physically most direct information extracted from such profiles is the location, accessibility, and structural character of configurations with small spin gaps, rather than an adiabatic spin–orbit-coupled free energy surface. When quantitative rates or state-to-state probabilities are required, the sampled crossing region configurations can be used as starting points for follow-up electronic structure calculations, including SOC evaluations or MECP/refined transition-state analyses.

### Spin-Gap Collective Variable

The central collective variable in SURFXsim is the spin gap

6 $$s_{sx}\left(r\right)=V_{B}\left(r\right)-V_{A}\left(r\right)$$

Equation 6 corresponds directly to the energy difference between the two spin states at the current nuclear configuration. By construction, this quantity is positive in regions where state *A* is more stable, negative in regions where state *B* is more stable, and zero at the crossing point (Figure 2). The spin-gap CV therefore provides a direct, physically interpretable descriptor of the progress toward the region where the two spin-state surfaces become energetically competitive.

An important advantage of this CV is that its derivatives can be written directly in terms of the state-specific forces

7 $$\frac{\partial s_{sx}}{\partial r_{i}}=\frac{\partial V_{B}}{\partial r_{i}}-\frac{\partial V_{A}}{\partial r_{i}}=-F_{B,i}+F_{A,i}$$

Equation 7 means that once the backend calculator provides the energies and forces for the two spin states, the spin-gap CV and its Cartesian derivatives are immediately available and can be passed to PLUMED without additional electronic structure approximations.

Because *s*_sx_ directly identifies the crossing region, it is particularly useful for distortion-driven spin crossover problems in which a single structural distortion modulates the state ordering. More generally, the CV can also be combined with additional coordinates, such as distances or coordination numbers, when the spin transition is coupled to a reactive event, in order to capture the relevant slow degrees of freedom and thereby reduce hysteresis along the reaction path and mitigate sampling imbalances. When an obvious structural parameter is known and is nearly one-to-one with the spin gap, biasing that structural coordinate and monitoring *s*_sx_ can be a valid and efficient alternative. Biasing *s*_sx_ directly is most advantageous when several geometric distortions contribute simultaneously, when no single structural descriptor is known in advance, or when the goal is specifically to sample all thermally accessible configurations with small diabatic spin gaps. In practice, comparing forward and backward passages in the *s*_sx_–structure space, together with time traces of key structural descriptors, provides a useful check for hysteresis and for deciding whether additional CVs are needed.

### Enhanced Sampling and Bias Forces

To drive rare spin-forbidden events, we combine the mixed Hamiltonian with metadynamics.^23^ In this approach, a history-dependent bias is deposited along one or more collective variables to discourage revisits to previously sampled regions and thereby accelerate exploration of the free-energy landscape. For a metadynamics bias acting on the spin-gap CV, the bias potential can be written formally as

8 $$V_{bias}\left(s_{sx},t\right)=\sum_{j=0}^{N\left(t\right)}\omega_{j}\;\exp\left[-\frac{{\left(s_{sx}-s_{sx}{,}_{j}\right)}^{2}}{2\sigma^{2}}\right]$$

In eq 8, ω_*j*_ and σ are the height and width of the deposited Gaussians, respectively, and *s*_sx_,_*j*_ is the CV value at deposition event *j*.

The corresponding bias force acting on atom *i* is

9 $$F_{i}^{bias}=-\frac{\partial V_{bias}}{\partial s_{sx}}\frac{\partial s_{sx}}{\partial r_{i}}$$

Substituting eqs 7 into [Disp-formula eq9] shows that the bias force is proportional to the difference between the forces of the two electronic states. This makes the implementation straightforward: SURFXsim writes state energies and forces to a file that is read by our custom PLUMED collective variable, which computes the spin gap and its derivatives, and returns the biasing forces to the molecular dynamics engine.

In the simplest use case, metadynamics is performed along the spin-gap coordinate alone. This is appropriate when the spin transition is tightly coupled to a single dominant structural distortion. However, because the implementation is fully compatible with multidimensional enhanced sampling, SURFXsim can also combine the spin-gap CV with a second coordinate, such as a bond distance, to describe reactive spin-forbidden events more faithfully and prevent hysteresis. Note that enhanced sampling is not formally required in every single application. If unbiased mixed-Hamiltonian dynamics already produces repeated reversible visits to both end-state basins and to the *s*_sx_ ≈ 0 region on the available time scale, metadynamics mainly provides a controlled route to accelerate sampling and reconstruct a free-energy profile. For larger barriers, slow solvent/ligand reorganizations, or reactive rearrangements, metadynamics is expected to be essential. We therefore recommend short unbiased pilot trajectories as a baseline diagnostic before production-enhanced sampling calculations.

## Results and Discussion

### Distortion-Driven Spin Crossover in [Fe­(2-pic)_3_]^2+^

We first considered a distortion-driven spin crossover in an [Fe­(2-pic)_3_]^2+^ model system, where the relative stability of the two spin states is strongly modulated by the Fe–N coordination environment,^28^ as illustrated in Figure 3a. This example is particularly well suited to testing the central premise of SURFXsim, namely, that the spin-gap CV alone can capture the relevant progress coordinate for a spin transition when the state ordering is controlled primarily by a structural distortion. The expected physical picture is straightforward. Shorter Fe–N distances favor the low-spin state, whereas elongated Fe–N distances stabilize the high-spin configuration. If the spin-gap CV is a meaningful descriptor of the transition, its evolution along the trajectory should be strongly correlated with the average Fe–N distance and with the mixing parameter λ. In particular, we expect the crossing region to be characterized by *s*_sx_ ≈ 0 and λ ≈ 0.5, while trajectories on either side of the crossing should show well-defined positive or negative spin gaps and λ values close to 1 or 0, respectively.

**Figure 3:**
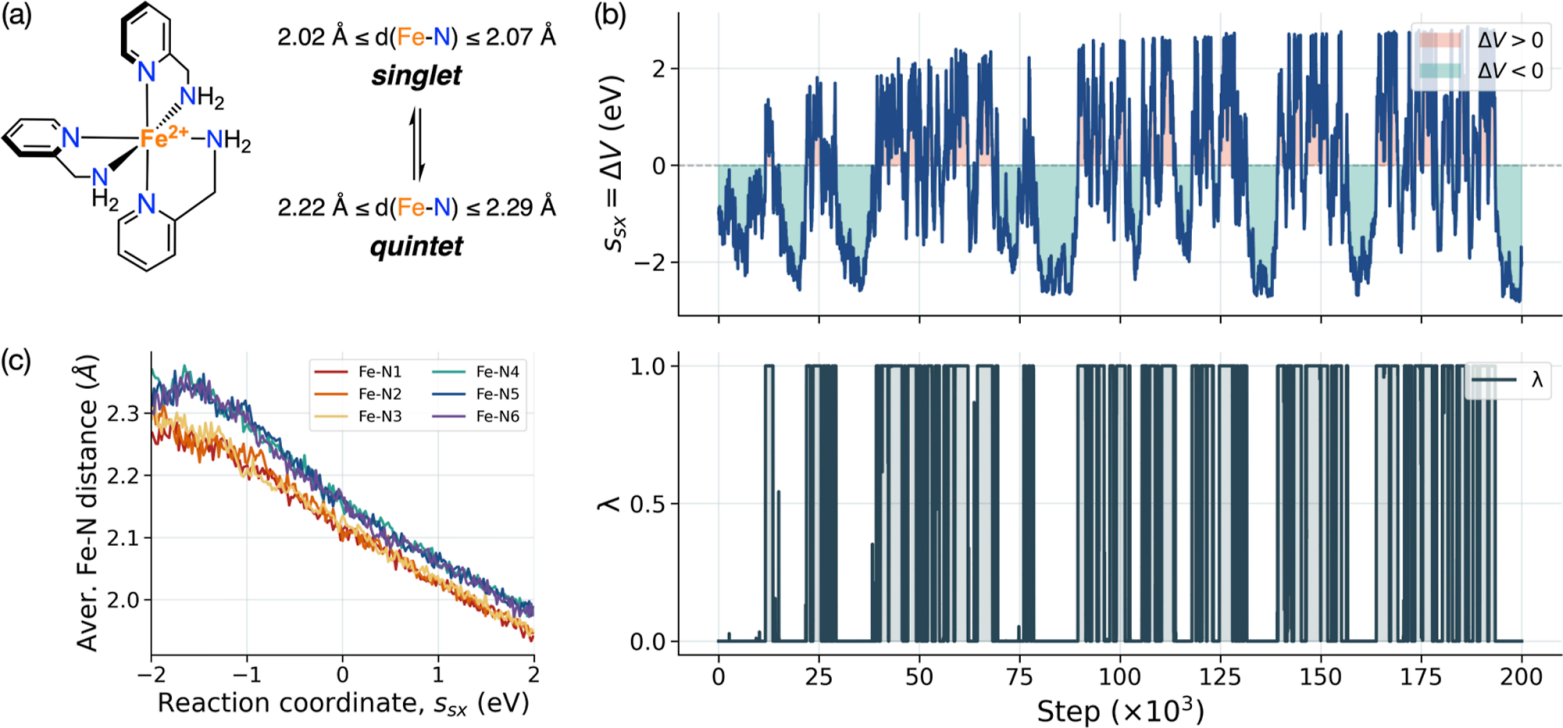
(a) Chemical structure of the [Fe­(2-pic)_3_]^2+^ complex, indicating the Fe–N bond-length ranges characteristic of the singlet and triplet states. (b) Time evolution of the collective variable *s*_sx_, i.e., Δ*V* (top), and of the switching parameter λ (bottom) during the metadynamics simulation, showing the correspondence between the sign of Δ*V* and the value of λ. (c) Average Fe–N bond distances as a function of *s*_sx_, illustrating the structural response of the Fe coordination sphere along the spin-bias coordinate.

The analysis of this system focuses on three complementary observables: the time evolution of the spin-gap CV, the corresponding evolution of the mixing parameter λ, and the variation of the Fe–N distances as key structural descriptors along the reaction coordinate. Figure 3b shows that the collective variable *s*_sx_ and the switching parameter λ evolve in a strongly correlated manner along the metadynamics trajectory: when Δ*V* becomes positive, λ approaches 1, whereas for negative Δ*V*, λ decreases toward 0. This behavior demonstrates that the switching function responds correctly to the sign of the spin-bias coordinate and smoothly connects the two spin-state end regions of the mixed Hamiltonian. Figure 3c further confirms that this interpolation is accompanied by the expected structural reorganization of the coordination sphere, as the averaged Fe–N distances vary systematically along the *s*_sx_ reaction coordinate. The switch from distinctly short to long Fe–N bond patterns across the coordinate is fully consistent with the known structural differences between low-spin and high-spin Fe­(II) configurations.^28^ The same coupling is visible directly in the time domain from the individual Fe–N distance traces reported in the Supporting Information (Figure S1). The evolution of the bias forces acting on atoms along the metadynamics trajectory (Figure S2) also demonstrates that bias potential accumulates correctly along the reaction coordinate defined by *s*_sx_. Taken together, these results provide direct evidence that SURFXsim drives the system to cross from one spin-state basin to the other rather than merely fluctuating around a single-state minimum. Of note, the multiple recrossing events observed along the trajectory further demonstrate reversibility and confirm that the method samples a genuine free-energy basin crossing rather than a single irreversible relaxation event.

Beyond the time (or step)-domain analysis, reconstruction of the free-energy surface from the accumulated bias potential provides direct access to the thermodynamic landscape along the spin-gap collective variable while also offering insight into the kinetics through the identification of barriers and crossing regions. At the same time, this framework enables a statistical characterization of the structural distortions sampled along the trajectory, particularly in the vicinity of the crossing region around *s*_sx_ ≈ 0, where the most relevant spin-switching events occur, thereby yielding valuable mechanistic insights into the transition. In the present case, correlating *s*_sx_ with the average Fe–N distances further demonstrates that the CV is not merely a numerical descriptor but is directly connected to the geometric reorganization governing the spin crossover. The corresponding free energy and structure–CV analyses are provided in Figure S3.

It is important to stress that this application is intended strictly as a proof-of-concept for the SURFXsim methodology rather than as a quantitative validation of the underlying machine-learning potential. In particular, the UMA model employed here (see the Computational Details section for further details) does not accurately reproduce the spin-state ordering reported in previous DFT studies for this system,^28^ and we therefore do not interpret the resulting state energetics or relative basin stabilities as quantitatively realistic. Instead, the purpose of this example is to assess whether SURFXsim can correctly drive and monitor a structurally induced spin-state transition using an inexpensive backend that explicitly distinguishes between spin manifolds. Overall, this first example aims to establish the simplest and most direct use case for SURFXsim: a distortion-driven spin crossover in which the spin-gap CV alone is sufficient to both identify the relevant transition pathway and drive sampling between the two spin states.

### Reactivity-Driven Spin Crossover in Fe­(CO)_*n*_

The second example addresses a more challenging scenario in which the spin transition does not only involve a subtle structural distortion within a molecule but also a reactive coordinate associated with a bond cleavage/formation event. For this purpose, we considered an archetypal spin-forbidden process: the coordination of a fifth CO ligand to Fe­(CO)_4_. While Fe­(CO)_4_ exhibits a triplet ground state, the coordination of an additional CO yields Fe­(CO)_5_, which stabilizes as a singlet.^29–32^ In such a case, the spin-gap CV remains essential because it directly identifies the crossing region, but it is not expected to be sufficient on its own to describe the full reaction coordinate. In particular, the spin-gap CV may vary only weakly across configurations in the unbound state, so that the resulting biasing forces are insufficient to drive the system back toward the bound state. This can lead to sampling imbalances and, ultimately, to hysteresis along the reaction path. Hence, in this case, the reactive event should also be resolved through a structural coordinate, here chosen as a metal–carbon distance. A coordination number would also be a natural choice for ligand association/dissociation. We used a single Fe–C distance here because the proof-of-concept setup follows one labeled incoming CO ligand and because this coordinate gives a transparent two-dimensional representation of the coupled spin-crossing/binding process. For fluxional systems with multiple equivalent ligands or exchange of the reactive ligand identity, a coordination-number CV would likely be preferred. From a methodological standpoint, this example highlights an important advantage of SURFXsim: the spin-gap CV is not limited to situations in which the spin gap alone acts as a complete reaction coordinate. Instead, it can be incorporated into a multidimensional enhanced-sampling setup in which the spin-gap CV captures the crossing aspect of the problem while additional coordinates describe the underlying chemistry, thus broadening the scope of the method.

Figure 4 compiles the main results of the two-dimensional metadynamics simulation. As in the previous example, the evolution of the spin-gap CV and the mixing parameter λ shows the expected physical correlation (Figure 4a). In addition, the second collective variable, *d*_Fe–C_, is also strongly correlated with the spin-gap CV, supporting the idea that the coordination number of the Fe center, or equivalently the Fe–C bond distance associated with ligand dissociation, is a meaningful descriptor of its preferred spin state. More specifically, decoordination of the fifth CO ligand from the Fe center, reflected in the elongation of the Fe–C distance (Figure 4a, purple line at the bottom), occurs after approximately 70,000 steps, as the system evolves from the singlet region (positive *s*_sx_ values and λ = 1) to the triplet region (negative *s*_sx_ values and λ = 0). Then, after filling the potential well corresponding to the unbound state, the ligand rebinds upon recrossing from the triplet to the singlet region at around 130,000 steps.

**Figure 4:**
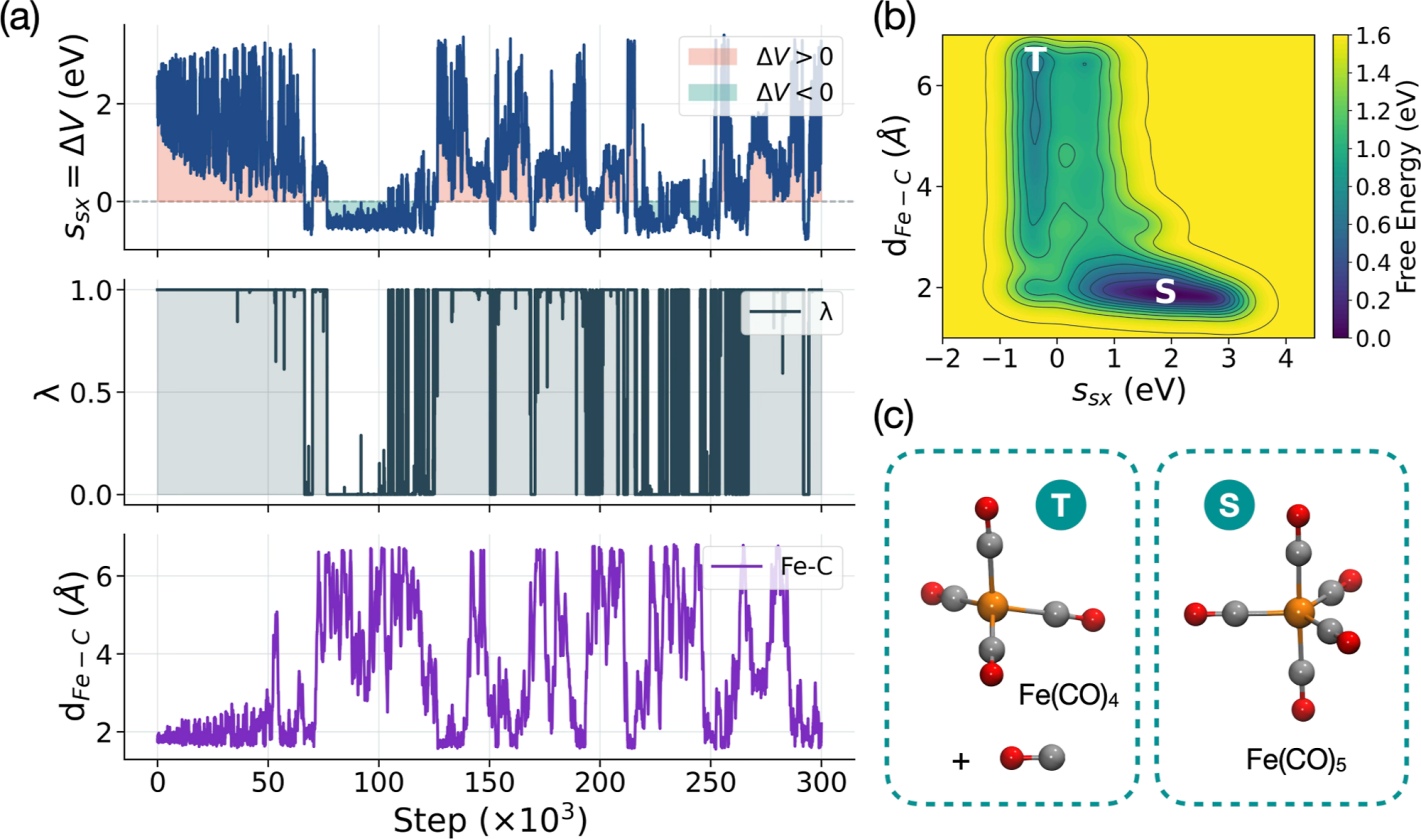
(a) Time evolution of the spin-gap collective variable *s*_sx_ (top), switching parameter λ (center), and Fe–C distance used as the second collective variable (bottom) during the two-dimensional metadynamics simulation of Fe­(CO)_*n*_ (*n* = 4, 5). (b) Reconstructed two-dimensional free-energy surface as a function of *s*_*sx*_ and the Fe–C distance, highlighting the minima associated with the triplet and singlet states, marked by white T and S, respectively. The contour interval is approximately 0.18 eV. (c) Representative structures corresponding to the two free-energy minima, illustrating the structural differences between the triplet and singlet regions of the surface.

A closer look to the plots reveals that changes in the bond distance and changes in spin-state character occur in a correlated but not necessarily simultaneous fashion. Throughout the simulation, the trajectory also samples configurations with long Fe–C distances in the singlet state and short Fe–C distances in the triplet state (see Figure S4 for a detailed representation of the trajectory onto the two-dimensional CV space). These configurations are associated with explorations into off-minimum-energy-path regions, highlighting the broader configurational diversity accessible during the spin-crossover process, as illustrated by the reconstructed free energy landscape of Figure 4b. When projected on the CV variable space, this free energy landscape allows identifying the singlet basin (denoted by a “S”), sitting at positive values of *s*_sx_ and short Fe–C distances; the triplet basin, denoted by a “T” at *s*_sx_ < 0 and long Fe–C distances; and the crossing region, at *s*_sx_ around zero. Representative configurations of both stable states are shown in Figure 4c.

In this example, for the energy surface provided by the employed MLP, the scattered Fe–C distances sampled in the crossing region (Figure S5) suggest that the two events underlying the overall process, namely spin-state change and Fe–CO bond formation, may occur either concomitantly or in a stepwise manner with similar energy costs. In the latter case, CO binding could first take place on the triplet surface, followed by intersystem crossing to the singlet, or alternatively, the system could undergo the crossing through thermal distortions before completion of the binding of the fifth CO ligand in the singlet state. A tentative minimum-free-energy path traced on this surface, together with representative crossing-region structures selected near *s*_sx_ = 0, is reported in the Supporting Information (Figure S6 and Tables S2–S4). These structures should be viewed as MECP-like configurations on the sampled mixed-Hamiltonian free-energy surface and as candidates for subsequent electronic-structure refinement, rather than as optimized MECP structures. In a broader sense, these observations should not be taken as definitive chemical conclusions, but rather as an illustration of the type of mechanistic insight that this analysis can provide, since the detailed shape of the free-energy surface is expected to depend on the underlying potential employed and has not been quantitatively benchmarked here against higher-level ab initio reference data. More generally, for any system of interest, visual inspection of the free-energy surface can provide a deeper understanding of how structural rearrangements and spin-state switching are coupled along the reaction pathway.

### DFT-Backed Distortion-Driven Intersystem Crossing in Methylcarbene

Finally, to demonstrate that SURFXsim is not restricted to machine-learning potentials, we interfaced the method with the Gaussian 16 quantum chemistry package^27^ through ASE and applied it to the distortion-driven singlet–triplet crossover of methylcarbene using a DFT potential. Methylcarbene (Figure 5a) stabilizes as a triplet with an equilibrium C–C–H angle of approximately 134° and undergoes a transition to the singlet state when the C–C–H angle narrows to about 106°.^33,34^ In this case, the spin-gap CV is strongly nonorthogonal to the angle. This system is a useful proof-of-principle example because the spin transition is strongly coupled to a simple structural distortion, making it conceptually similar to the [Fe­(2-pic)_3_]^2+^ case while relying on a conventional electronic-structure backend.

**Figure 5:**
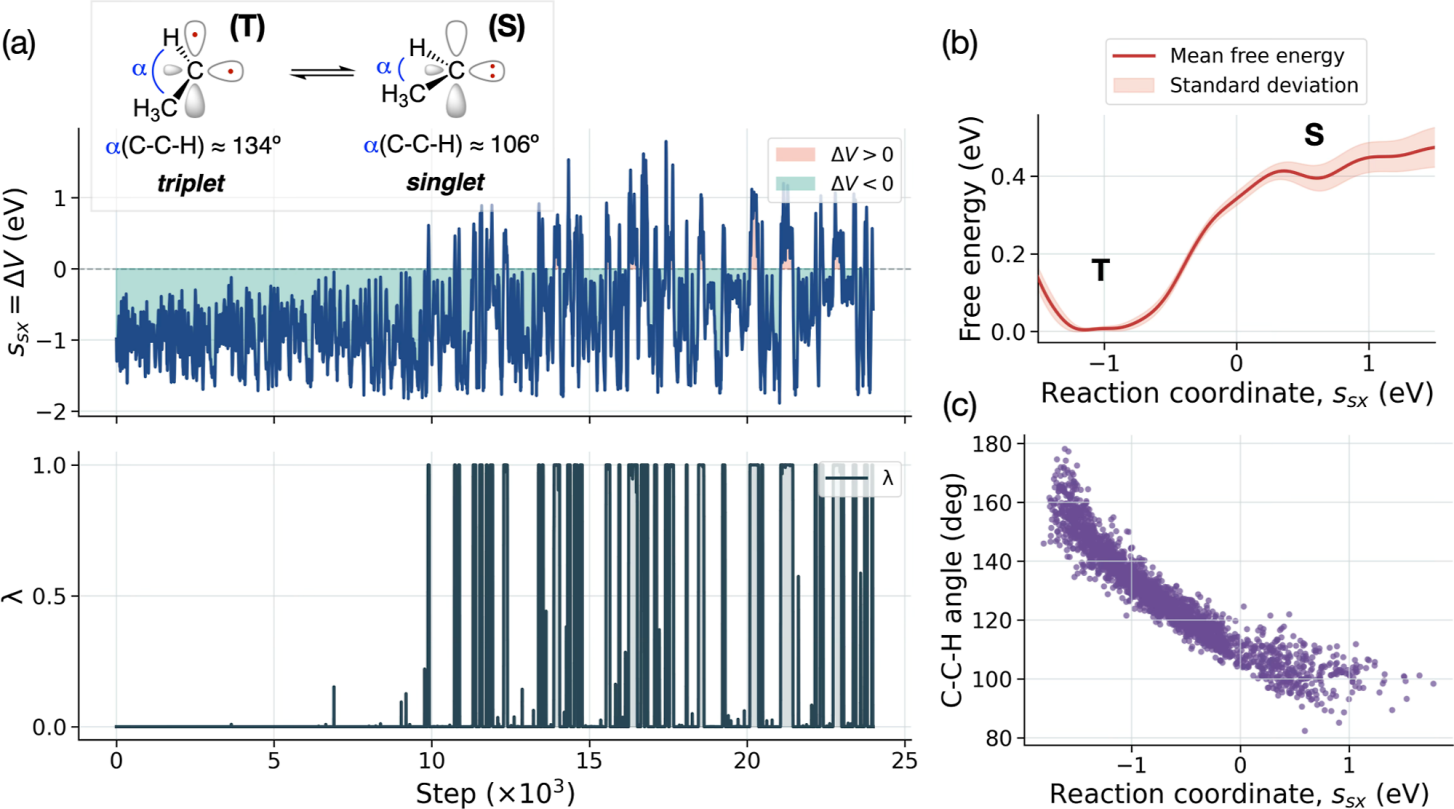
(a) Chemical structure of methylcarbene, highlighting the angle variation coupled with intersystem crossing between singlet and triplet states. The graphs show the evolution of the collective variable *s*_sx_ (top) and of the switching parameter λ (bottom) during the DFT-MD metadynamics simulation. (b) Reconstructed free energy surface averaged over the last 100 Gaussian kernel depositions, indicating triplet and singlet state basins with T and S, respectively. (c) Scatter plot of the measured C–C–H angles in the metadynamics trajectory as a function of the *s*_sx_ value.

Methylcarbene was simulated in vacuum using the BLYP GGA functional in combination with the small Pople-type 6-31G basis set in order to accelerate sampling. Note that, in the present work, this Gaussian-backed application is not intended as a production-level benchmark but rather as a demonstration of the generality of the method. The purpose of this section is therefore to show that the same SURFXsim framework, including the mixed Hamiltonian formalism and the spin-gap CV machinery, can be applied without conceptual modification when the underlying energies and forces are obtained from a DFT calculator rather than from a machine-learning potential. As shown in Figure 5a, during 24,000 steps of DFT-MD simulation coupled with metadynamics, the system undergoes several surface-crossing events, as reflected by the changes in sign of the spin-gap CV and the evolution of λ, which adapts the relative contribution of each state to the mixed Hamiltonian according to the instantaneous position along the reaction coordinate. The rapid recrossings from the singlet to the triplet state are consistent with the metastable character of the singlet state inferred from the free-energy profile in Figure 5b. The singlet-side minimum is shallow in this short DFT-backed proof-of-principle trajectory. This is qualitatively consistent with the known preference of methylcarbene for the triplet state, but we do not regard the depth of this well as quantitatively converged because the Gaussian-backed simulation was stopped once several recrossings had demonstrated portability of the implementation. Finally, Figure 5c shows that the spin-gap CV is able to capture the atomic rearrangements involved in the spin-crossover process, which in this case are strongly reflected in the C–C–H angle. Accordingly, this angle gradually decreases as the system evolves from the triplet region (*s*_sx_ < 0) to the singlet region (*s*_sx_ > 0). Unbiased baseline data and an explicit time trace of the C–C–H angle are provided in the Supporting Information (Figures S7 and S8) to further illustrate the correlation between *s*_sx_ and the structural proxy and to help separate spontaneous fluctuations from metadynamics-driven sampling. At the same time, because the biasing forces act on all atoms, the spin-gap CV is not restricted to a single geometrical parameter but is instead able to capture the broader set of atomic displacements that drive the system between spin states through a manifold of crossing configurations accessible by thermal disorder. As an additional proof-of-principle test, we performed a mixed-Hamiltonian simulation in which the C–C–H angle, rather than *s*_sx_, was used as the biased CV. The resulting structural distortion was sufficient to induce spin crossings, as confirmed by the concomitant evolution of *s*_sx_ and λ during the simulation (Figure S9). Nevertheless, directly biasing *s*_sx_ may be advantageous because, as stated above, it targets the energetic degeneracy between the spin states without assuming a single structural coordinate, thereby helping to reduce pathway-dependent hysteresis when similar angles correspond to configurations with different spin gaps.

## Conclusions

We have presented SURFXsim, a practical framework for simulating spin-forbidden events as finite-temperature rare processes on atomistic free energy landscapes. The method combines a configuration-dependent mixed Hamiltonian, a physically transparent spin-gap collective variable, and metadynamics-based enhanced sampling implemented through PLUMED. In this way, SURFXsim bridges an important gap between static crossing point analysis and computationally demanding nonadiabatic dynamics.

The examples considered here illustrate the versatility of this approach. For a distortion-driven spin crossover in [Fe­(2-pic)_3_]^2+^, the spin-gap CV alone is expected to provide a chemically meaningful and efficient descriptor of the transition. For a reactivity-driven iron carbonyl problem, the same CV can be combined with an additional structural coordinate in two-dimensional metadynamics, allowing the coupling between chemical reactivity and spin-crossover to be resolved. Finally, the methylcarbene example demonstrates that the implementation is not tied to a single backend and can be interfaced with conventional quantum chemistry codes through ASE.

Taken together, these results suggest that SURFXsim can become a useful tool for exploring spin-forbidden chemistry in systems that are too large or too slow for conventional nonadiabatic dynamics. At the same time, the reliability of the resulting crossing region structures and free energy barriers is necessarily limited by the quality of the two underlying spin-state potentials. This point is particularly important when using machine-learning potentials trained primarily on ground-state data or density functional approximations for systems where self-interaction error or spin-state energetics are problematic. We therefore view the spin-gap CV not only as a production-enhanced sampling coordinate but also as an exploratory coordinate that can identify candidate crossing configurations for subsequent refinement with higher-level electronic structure methods, SOC calculations, MECP optimizations, or more accurate free energy calculations. Looking ahead, several developments appear to be especially promising. These include interfaces to additional quantum chemistry packages, extensions to periodic density functional theory workflows, systematic applications to catalysis and materials chemistry, and broader use of multidimensional enhanced sampling protocols. We anticipate that such developments will further expand the range of spin-forbidden processes that can be studied at an atomistic resolution with affordable computational models.

## Computational Details

### Software Implementation

SURFXsim is implemented as a set of Python drivers that use ASE as the molecular simulation layer and PLUMED as the enhanced-sampling engine. The current repository on GitHub (https://github.com/sole-daura-lab/SURFXsim) includes three main workflows.

•A UMA-based implementation that retrieves the machine learning model online,
•A UMA-based implementation for offline use on clusters or restricted environments, and
•A Gaussian-based implementation using the ASE Gaussian calculator.

As shown in Figure 1, at each molecular dynamics step, the code evaluates state-specific energies and forces, computes Δ*V* and λ, and writes the information needed by PLUMED to a text file. A custom PLUMED module (SPINGAP.cpp) then computes the spin-gap CV and its derivatives. The Python driver subsequently propagates the trajectory with the mixed forces and records the time evolution of the relevant observables, including the spin gap, the mixing parameter, and structural descriptors.

The computational cost of a SURFXsim step is dominated by the two state-specific energy/force evaluations required to build Δ*V*, λ, and the mixed forces. Relative to a single-state unbiased MD simulation with the same backend, the leading cost is therefore approximately a factor of 2 before considering enhanced sampling. Since the two state-specific calculations are independent, this factor-of-two increase has trivial parallel scaling and can be offset by running both evaluations concurrently with additional computational resources. The additional PLUMED/file-I/O layer scales linearly with the number of atoms because the state-specific force arrays are written and read at each step. In our current Python implementation this overhead is small compared with DFT force evaluations, but it can become visible for very fast machine-learning backends or on file systems with slow metadata operations. For larger systems, the recommended optimization is to keep the spin-gap data exchange local to the compute node or to replace the text-file exchange by an in-memory interface in future implementations.

The modularity of the implementation is important. Any ASE-compatible calculator that can provide energies and forces for two spin states can, in principle, be interfaced with SURFXsim. In this work, we focus on machine learning and Gaussian backends, but the same design should be transferable to other quantum-chemistry codes and, in future work, to periodic density functional theory workflows.

### Systems and Simulation Strategy

We considered three proof-of-concept applications of increasing complexity. The first is a distortion-driven spin crossover between low-spin singlet and high-spin quintet states in a [Fe­(2-pic)_3_]^2+^ model system (49 atoms), which is used to demonstrate that the spin-gap CV alone can drive a structural spin transition. The second is a reactivity-driven spin-forbidden process in an iron carbonyl system, Fe­(CO)_4_, which has a triplet ground state but stabilizes as a singlet state upon coordination of a fifth CO ligand (Fe­(CO)_5_, 11 atoms). In this case, the spin-gap CV is combined with a Fe–C distance in a two-dimensional metadynamics protocol. The third is the distortion-driven singlet–triplet crossover of methylcarbene (6 atoms), which is used to demonstrate compatibility with a DFT potential through a Gaussian backend.

For all simulations, the two electronic states of interest were evaluated independently at each step, and the mixed-Hamiltonian dynamics were propagated in the canonical ensemble using a Langevin thermostat.^35^ Prior to dynamics, the structure was centered in the simulation cell and energy-minimized with the BFGS algorithm until the maximum force was below 0.05 eV Å^–1^. Thermal equilibration and production dynamics were performed in the canonical (*NVT*) ensemble using Langevin dynamics at 300 K with a friction coefficient of 0.05 atomic units and a time step of 0.5 fs. Initial velocities were assigned from a Maxwell–Boltzmann distribution at 300 K. An initial *NVT* equilibration run of 2000 steps was performed before metadynamics. The subsequent production metadynamics simulation was propagated for 200,000 MD steps in the case of [Fe­(2-pic)_3_]^2+^, 300,000 for the Fe­(CO)_*n*_ system, or 24,000 MD steps in the case of methylcarbene using a DFT potential. Metadynamics was performed through the PLUMED interface, through which the spin-gap CV was implemented as a custom PLUMED module that reads a text file containing the energies and forces for the two spin states and returns both the scalar CV value and its derivatives with respect to all Cartesian coordinates. Notably, in the present examples, enhanced sampling is required because the surface-crossing barriers exceed 0.4 eV, which is certainly too high to be overcome on the simulation time scale. Accordingly, unbiased mixed-Hamiltonian trajectories with a comparable setup are provided as a baseline in Figures S7 and S10, illustrating that the crossing region is not sampled in the absence of metadynamics. Trajectory frames were written every 10 steps. The simulations were stopped after repeated passages between the two end-state regions and after the reconstructed profiles no longer changed qualitatively over the final set of Gaussian depositions used for averaging. For future production applications, we recommend monitoring recrossing statistics, the stability of the reconstructed free-energy profile over successive trajectory blocks, and the absence of systematic hysteresis in the *s*_sx_–structure projection.

The metadynamics parameters used here were selected conservatively for proof-of-concept sampling. Gaussian widths were chosen to be comparable to the short-time fluctuations of each CV during the initial equilibration/pilot dynamics, while the hill height of 0.01 eV was kept below *k*_B_ *T* at 300 K to perturb the dynamics gently and avoid abrupt steering across the crossing region. Wall positions were placed outside the range needed to sample both end-state basins and the *s*_sx_ ≈ 0 region but inside values that led to unproductive excursions or chemically unreasonable geometries for the chosen backend potential. Plain metadynamics was used because the goal of these demonstrations was to force repeated recrossings and visualize the accessible landscape with a simple protocol. Well-tempered metadynamics should be preferable for quantitatively converged free-energy estimates in future applications, especially for larger barriers or longer multidimensional simulations.

### UMA-Based Simulations

The proof-of-concept simulations reported here for [Fe­(2-pic)_3_]^2+^ and Fe­(CO)_*n*_ systems use the UMA machine-learning potential through the FairChem/ASE interface^24,26,36^ (see the SURFXsim GitHub for further details on the employed versions of all Python libraries). In this setup, the code performs two state-specific energy and force evaluations at each step and combines the resulting magnitudes by means of the mixed Hamiltonian described above. This backend was chosen because it offers a computationally efficient route to repeated state evaluations, making it particularly suitable for long metadynamics trajectories. The specific UMA model used in this work was the uma-s-1p1 model with the “omol” task setting for molecular systems, which has been parametrized to account for distinct spin states.

The [Fe­(2-pic)_3_]^2+^ system was placed in a cubic cell of 20 × 20 × 20 Å^3^ with periodic boundary conditions applied in all directions, and the mixed Hamiltonian was constructed from two spin states evaluated on-the-fly, using charge +2 with spin multiplicities 1 and 5 for states A and B, respectively. Metadynamics was performed using the spin-gap CV with Gaussian hills of height 0.01 eV and width σ = 0.15 eV deposited every 100 steps. Upper and lower restraining walls were applied at *s*_sx_ = 2.5 and −2.5 eV, respectively, with a force constant of 5.0 eV. Note that for this test case, however, the uma-s-1p1 model does not reproduce the spin-state ordering reported in previous DFT studies,^28^ instead predicting the quintet state to be the most stable one. Accordingly, the UMA-based simulations reported here are not intended as quantitatively predictive calculations for this complex. Rather, the MLP is used as a low-cost proof-of-concept backend that provides access to multiple spin states and allows us to test the methodological machinery of SURFXsim, namely, the mixed-Hamiltonian propagation, the spin-gap collective variable, and the enhanced-sampling protocol.

For the Fe-carbonyl system, the Fe­(CO)_5_ system was also placed in a 3D-periodic cubic cell of 20 × 20 × 20 Å^3^ with periodic boundary conditions applied in all directions, and the structure was centered in the cell prior to dynamics. The mixed Hamiltonian was constructed from two spin states evaluated on-the-fly with the same underlying machine-learning potential, using charge 0 and spin multiplicities 1 and 3 for the singlet and triplet states, respectively. In this case, two-dimensional metadynamics was performed through the PLUMED interface using the spin-gap CV and the Fe–C distance for a randomly selected CO ligand as collective variables. Gaussian hills of height 0.01 eV were deposited every 100 steps with widths σ = 0.25 eV for the spin-gap CV and 0.2 Å for the Fe–C distance. Upper restraining walls were applied to the spin-gap CV at +3.5 eV and to the five Fe–C distances at 6.5, 2.8, 2.8, 2.8, and 2.8, respectively (the former corresponding to the reactive CO), with a force constant of 5.0 eV, while a lower wall was applied to the spin-gap CV at −4.0 with the same wall strength.

### Gaussian-Based Simulations

To demonstrate the portability of SURFXsim beyond machine-learning potentials, we also interfaced the code with Gaussian 16 (rev. A.03) through ASE.^24,27^ In this case, the two spin states are evaluated through separate single-point DFT calculations at each step, and the resulting energies and forces are passed to the same mixed-Hamiltonian and PLUMED machinery described above. In the current implementation, this workflow was demonstrated for methylcarbene using a BLYP/6-31G setup.^37–39^ As Gaussian does not support periodic calculations, this system was simulated in vacuum. The mixed Hamiltonian was built up from two states with a charge of zero and spin multiplicities of 1 and 3 for states A and B, respectively. Metadynamics was carried out using *s*_sx_ as the sole CV and spawning Gaussian hills of height 0.01 eV and width σ = 0.15 eV every 100 steps. Repulsive upper and lower walls were applied to the spin-gap collective variable at +3.0 and −3.0 eV, respectively, using a wall strength of 5 eV.

## Supplementary Material



## Notes

Repository overview and file-purpose table; time evolution of the six Fe–N distances and their average during the [Fe­(2-pic)_3_]^2+^ metadynamics trajectory; RMS bias-force evolution for the same trajectory; reconstructed [Fe­(2-pic)_3_]^2+^ free-energy surface and a representative minimum-energy-crossing-region structure; Fe­(CO)_*n*_ trajectory projected on the two-dimensional spin gap/Fe–C-distance collective-variable space; distribution of Fe–C distances sampled near *s*_sx_ = 0; short- and long-Fe–C minimum-free-energy pathways for Fe­(CO)_*n*_, their free-energy profiles, and representative crossing-region configurations; Fe–C distances, angles involving the incoming CO ligand, and internal C–Fe–C angles for those configurations; unbiased methylcarbene baseline trajectory reporting *s*_sx_, λ, and the C–C–H angle; time evolution of the methylcarbene C–C–H angle during *s*_sx_-biased metadynamics; evolution of the C–C–H angle, *s*_sx_, and λ during a test metadynamics simulation in which the angle is used as the biased collective variable; and unbiased [Fe­(2-pic)_3_]^2+^ baseline trajectory reporting *s*_sx_, λ, and the average Fe–N distance (PDF)

## Abbreviations

ASE: atomic simulation environment
MD: molecular dynamics
MECP: minimum-energy crossing point
CV: collective variable
PES: potential energy surface
MLP: machine-learning potential
SOC: spin–orbit coupling
