# Supplementary material for: SURFXsim: Free-Energy Simulations of Spin-Forbidden Events with a Spin-Gap Collective Variable
Source: J Chem Theory Comput. 2026 Jul 25;22(15):7512–22. doi: 10.1021/acs.jctc.6c00796 (PMC13471722; doi:10.1021/acs.jctc.6c00796)
Supplement: Supplementary file 1 [file ct6c00796_si_001.pdf]

Supporting Information

SURFXsim: Free-Energy Simulations of Spin-Forbidden Events with a Spin-Gap Collective Variable

Ajmal Rahman Mullukkandy[1,2] Bernd Ensing[3] Carles Bo[1,2] Albert Solé-Daura[1,2]*

[1] Universitat Rovira i Virgili, Department of Physical and Inorganic Chemistry, Marcel·lí Domingo, 1, 43007 Tarragona, Spain.

[2] Institute of Chemical Research of Catalonia (ICIQ-CERCA), The Barcelona Institute of Science and Technology, Avgda. Països Catalans, 16, 43007 Tarragona, Spain.

[3] Van 't Hoff Institute for Molecular Sciences and Amsterdam Center for Multiscale Modeling, University of Amsterdam, Science Park 904, 1098 XH Amsterdam, The Netherlands.

Email: albert.soled@urv.cat — asole@iciq.es

S1 Overview of the Repository

The current SURFXsim-related repository (https://github.com/sole-daura-lab/SURFXsim) contains Python drivers for UMA-based and Gaussian-based workflows together with a custom PLUMED collective variable implementation. In the present study, the main manuscript focuses on the methodological concept and proof-of-principle applications. The repository contains the main files compiled in Table S1, in addition to two examples on the use of SURFXsim for UMA- and Gaussian-based simulations.

Table S1: Summary of the main files included in the GitHub repository.

File	Purpose
`SURFXsim_UMA-online.py`	UMA-based workflow using an online model source
`SURFXsim_UMA-offline.py`	UMA-based workflow using local model files
`SURFXsim_Gaussian.py`	Gaussian-backed DFT workflow
`SPINGAP.cpp`	PLUMED collective variable for the spin gap
`README.md`	README file containing details about the code, its mathematical background, functioning, usage instructions and dependencies.

S2 Additional Analyses and Simulation Results

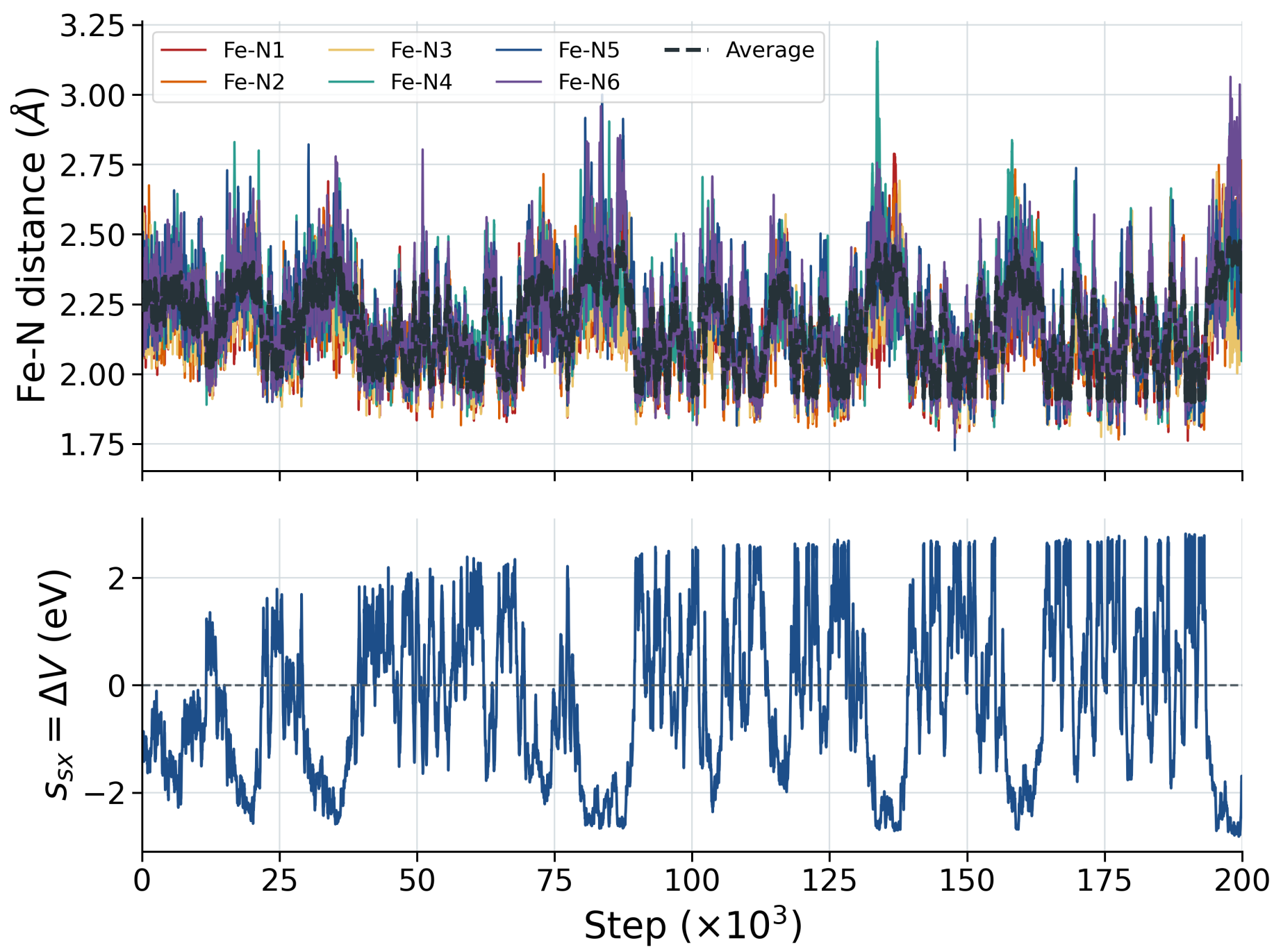

Figure S1: Time evolution of Fe–N distances during the $[Fe(2\text{-pic})_3]^{2+}$ metadynamics trajectory. This analysis complements Figure 3b of the main text by showing directly that the spin-gap CV drives the expected coordination-sphere reorganization.

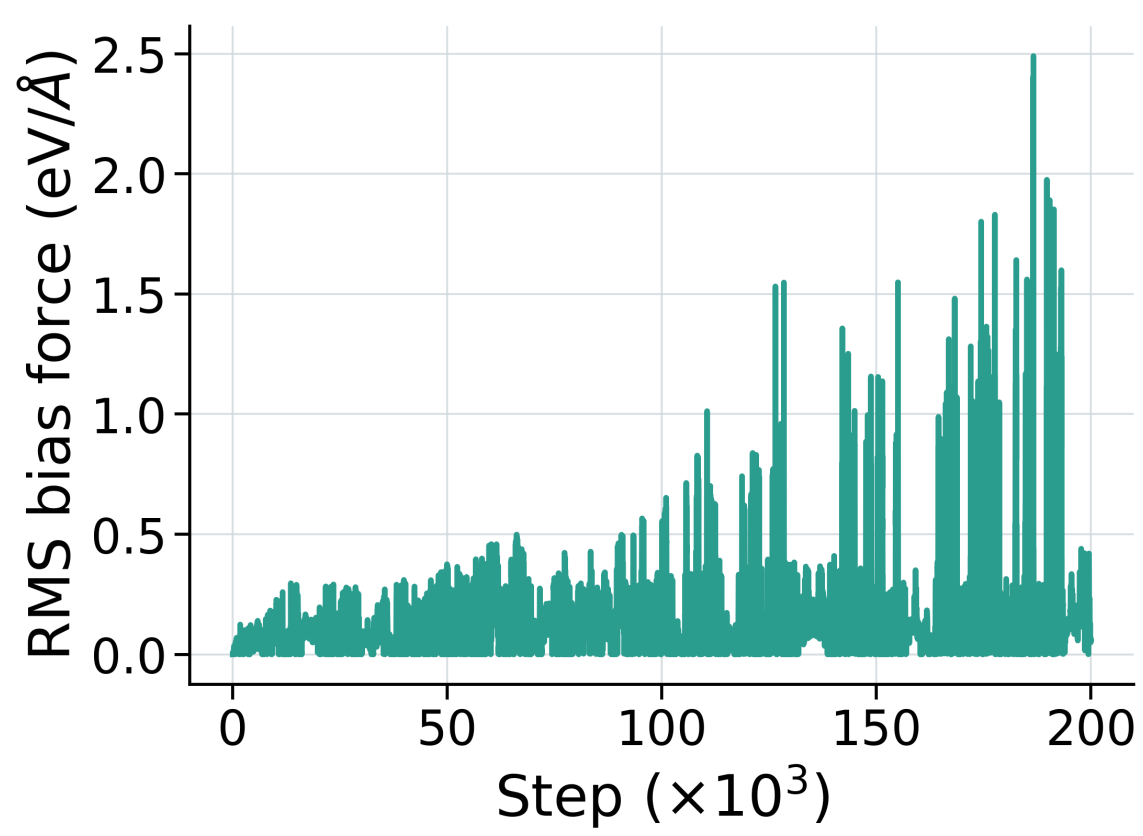

Figure S2: Evolution of the Root Mean Squared (RMS) bias forces along the metadynamics simulation with $[Fe(2\text{-pic})_3]^{2+}$.

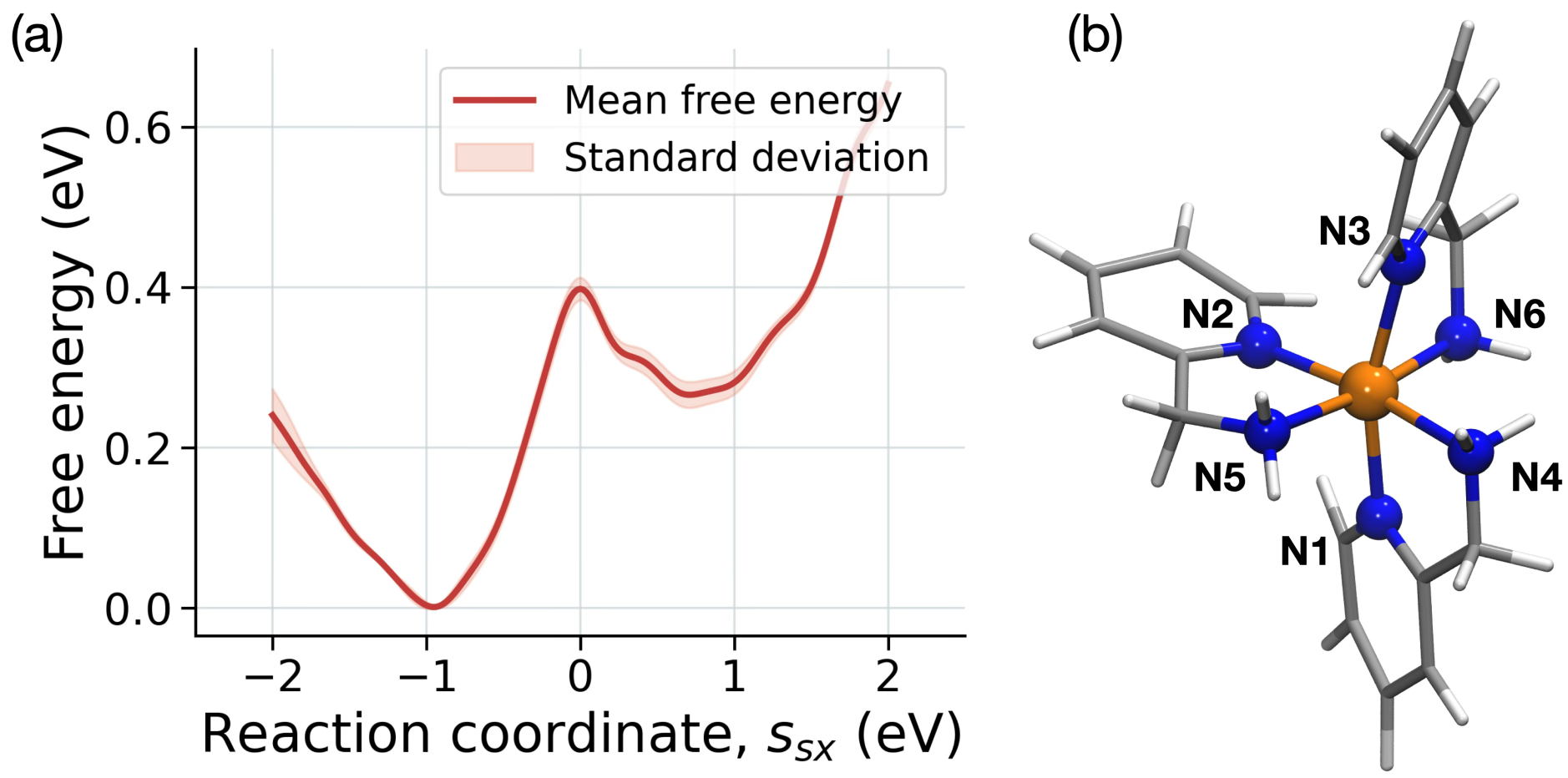

atom	d(Fe-N) (Å)
N1	2.095 ± 0.073
N2	2.108 ± 0.071
N3	2.134 ± 0.063
N4	2.137 ± 0.053
N5	2.158 ± 0.063
N6	2.179 ± 0.058

Figure S3: (a) Reconstructed free energy surface for the spin crossover simulation of $[Fe(2\text{-pic})_3]^{2+}$, averaged over the last 100 Gaussian kernel depositions. (b) Representative structure of the MECP, obained from the snapshot of the trajectory with the smallest absolute value of s_{sx} ($2.82{\cdot}10^{-4}$ eV). Key Fe-N distances (Å) are averaged over all trajectory frames with s_{sx} values between -0.005 and +0.005 eV.

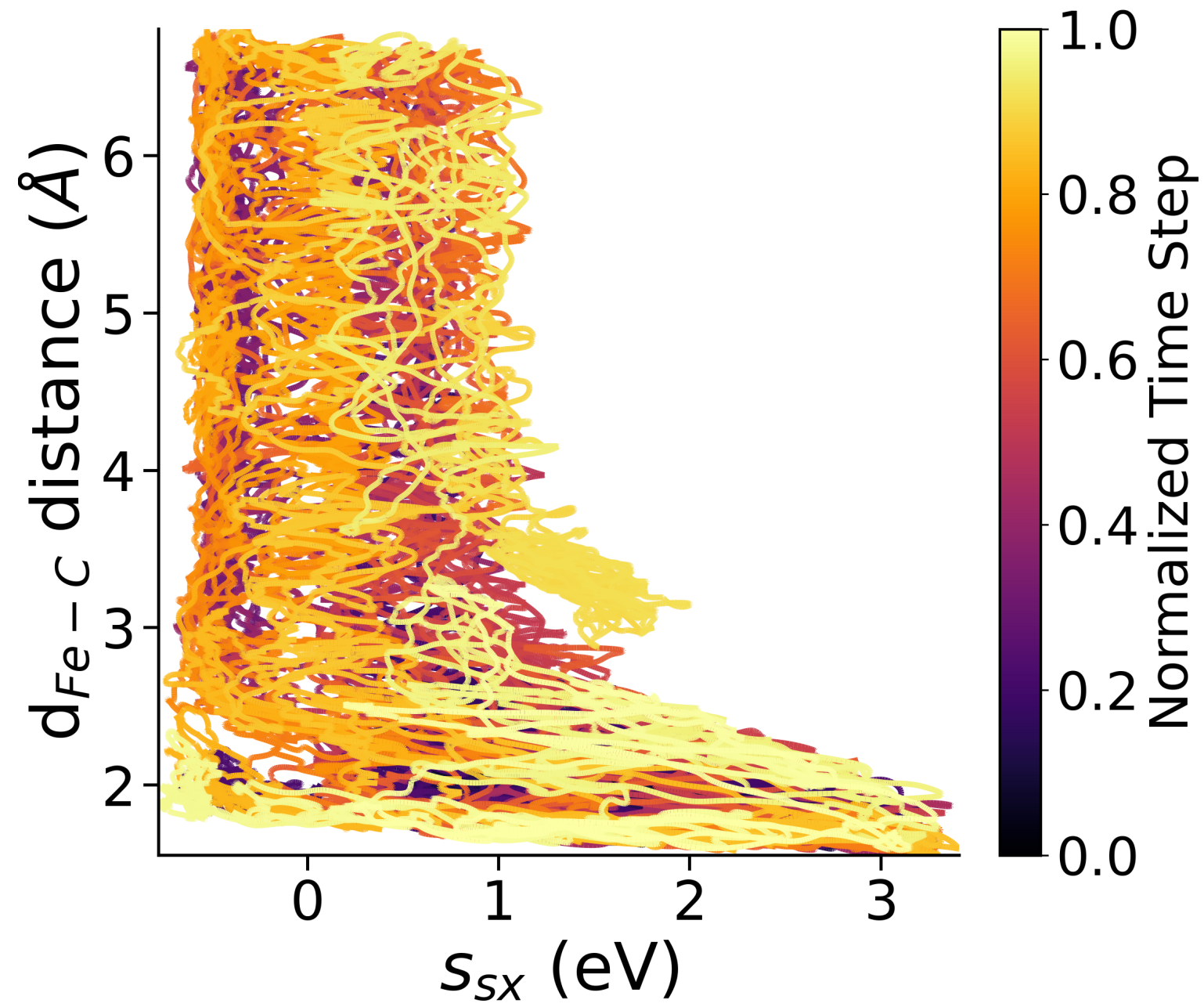

Figure S4: Trajectory of the $Fe(CO)_n$ metadynamics represented on the two dimensional CV space and colored by time.

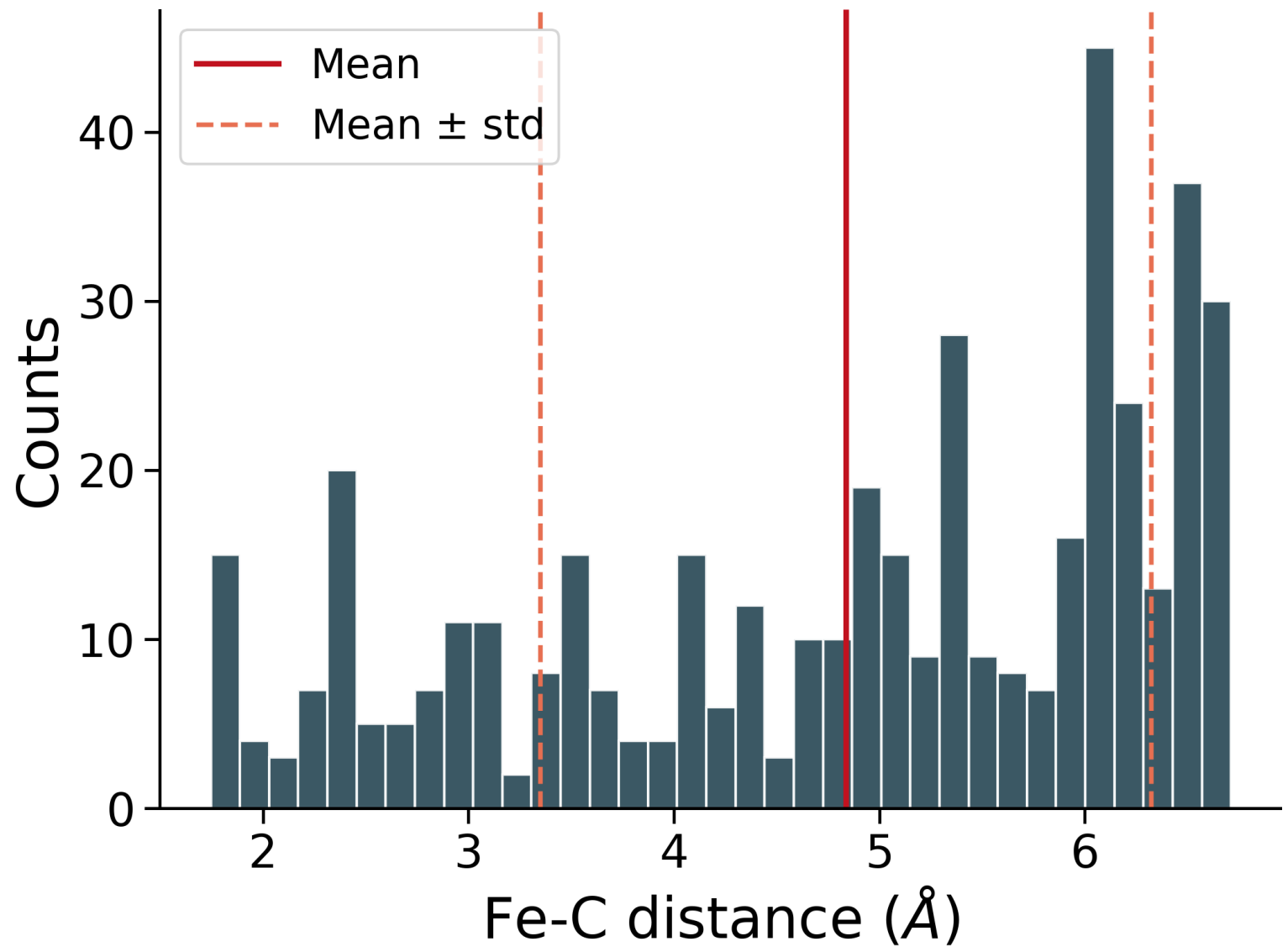

Figure S5: Histogram of the CV2 (Fe–C distance) values near the crossing region; that is, at CV1 (s_{sx}) close to zero (range from -0.005 to +0.005 eV).

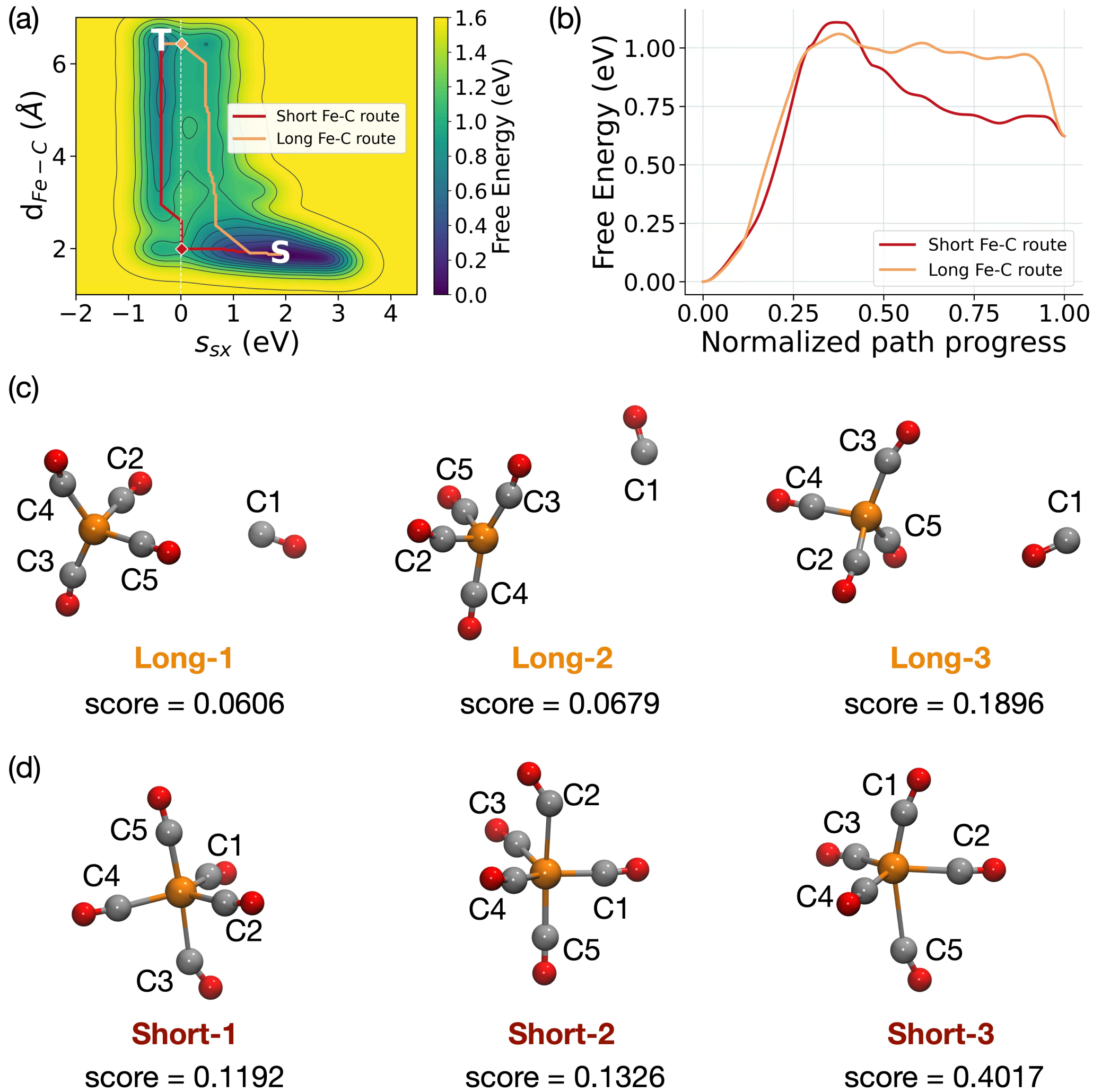

Figure S6: Analysis of the two crossing pathways identified for $Fe(CO)_n$. (a) Reconstructed two-dimensional free-energy surface as a function of s_{sx} and the Fe–C1 distance, showing the short-distance (red) and long-distance (orange) minimum-free-energy pathways connecting the singlet (**S**) and triplet (**T**) basins. Diamonds indicate the crossing points nearest $s_{sx} = 0$ for each pathway, while the dashed line marks $s_{sx} = 0$. (b) Free-energy profiles along the normalized progress coordinate of the two pathways. (c,d) Three highest-ranked representative configurations from the long- and short-distance crossing channels, respectively. Representative crossing-region structures were selected from trajectory frames satisfying $|s_{sx}| \leq 0.02$ eV. Frames were then classified into short- and long-distance channels using Fe–C ranges of 1.6–3.2 Å and 4.0–6.8 Å, respectively. Within each channel, structures were ranked using the score $(s_{sx}/0.02)^2 + [(d_{\text{Fe–C}} - d_{\text{target}})/0.30]^2$, where d_{target} was 1.99 Å for the short-distance pathway and 6.43 Å for the long-distance pathway, as determined from the corresponding minimum-free-energy paths; and 0.02 eV and 0.3 Å serve as characteristic energy and distance tolerances used to normalize deviations, so that they both contribute equaly to the score. The three highest-ranked structures from each channel were retained, requiring a minimum separation of 500 simulation steps to avoid selecting closely related consecutive configurations. C1 denotes the incoming CO carbon, whereas C2–C5 denote the four carbonyl carbons of the $Fe(CO)_4$ fragment. The corresponding Fe–C distances and C–Fe–C angles are reported in Tables S2-S4.

Table S2: Fe–C distances for the representative short- and long-distance crossing structures. C1 denotes the incoming CO carbon, while C2–C5 denote the four carbonyl carbons initially coordinated to Fe.

Structure	Rank	Step	s_{sx} (eV)	Fe–C1	Fe–C2	Fe–C3	Fe–C4	Fe–C5
				Distance (Å)				
Short	1	189340	-0.0052	1.922	2.090	2.337	2.049	1.991
Short	2	294360	0.0032	1.892	2.392	1.987	1.875	2.074
Short	3	250080	0.0067	1.828	2.094	2.061	1.959	2.736
Long	1	229990	-0.0041	6.471	1.821	1.871	1.924	1.859
Long	2	199950	0.0014	6.355	1.800	1.778	1.871	1.866
Long	3	233530	0.0070	6.507	1.806	1.943	1.767	1.927

Table S3: Angles involving the incoming CO carbon C1 for the representative crossing structures. For the long-distance channel, these values describe the orientation of the separated incoming CO relative to the $Fe(CO)_4$ fragment.

Structure	Rank	C1–Fe–C2	C1–Fe–C3	C1–Fe–C4	C1–Fe–C5
		Angle (°)			
Short	1	111.3	83.8	125.0	96.1
Short	2	91.5	105.7	134.7	87.4
Short	3	82.6	98.7	103.1	162.0
Long	1	38.1	88.8	132.9	68.5
Long	2	149.4	58.2	114.2	73.1
Long	3	88.2	73.9	175.8	76.9

Table S4: Internal C–Fe–C angles of the $Fe(CO)_4$ fragment for the representative short- and long-distance crossing structures.

Structure	Rank	C2–Fe–C3	C2–Fe–C4	C2–Fe–C5	C3–Fe–C4	C3–Fe–C5	C4–Fe–C5
		Angle (°)					
Short	1	91.0	122.6	93.2	85.1	175.6	91.4
Short	2	83.4	78.9	172.4	116.8	104.2	96.5
Short	3	122.2	119.1	79.5	116.8	88.9	87.8
Long	1	100.7	99.3	97.3	127.1	112.2	113.1
Long	2	91.4	95.1	112.8	156.8	99.8	98.2
Long	3	130.3	93.3	104.2	102.2	115.6	106.4

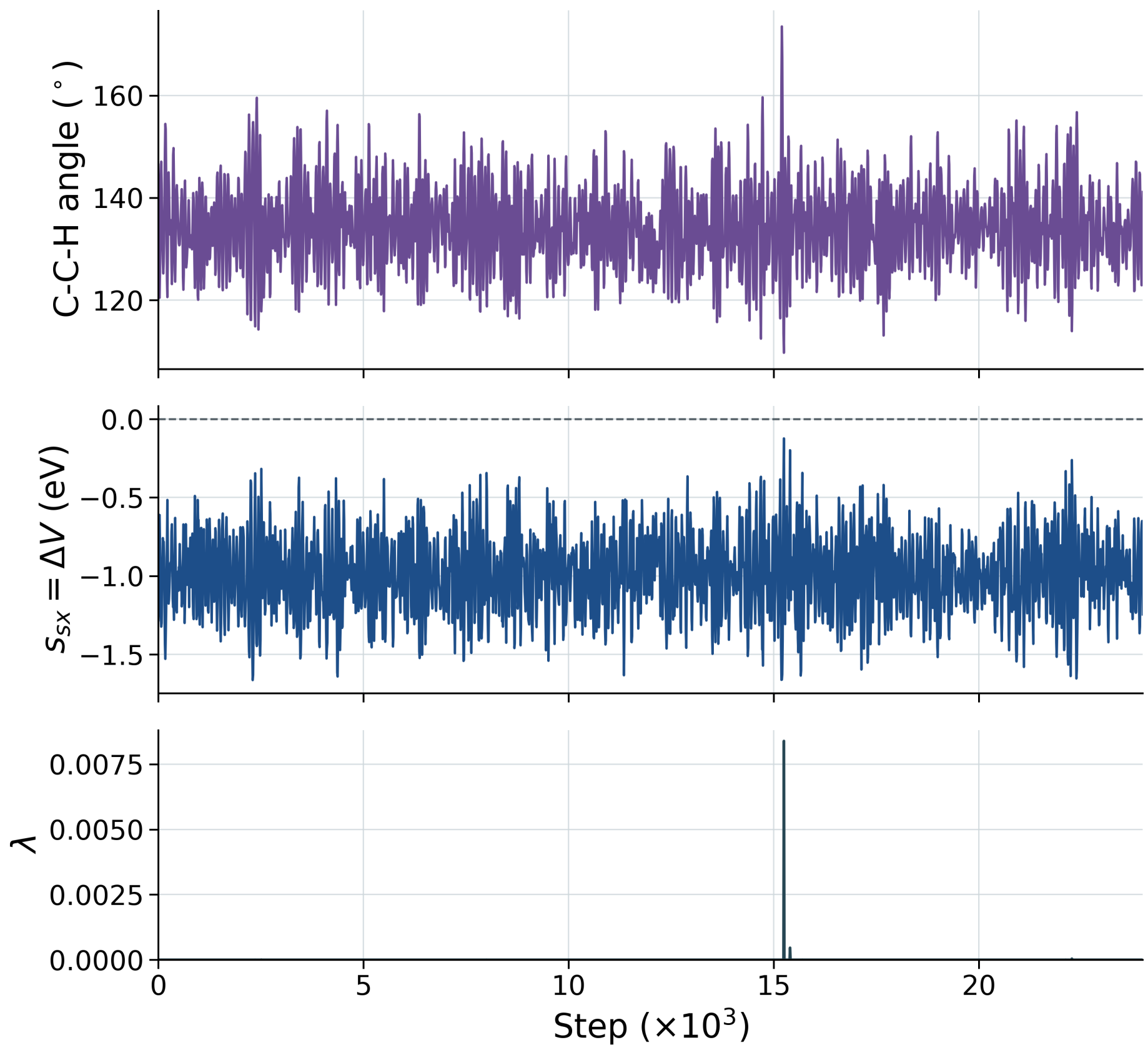

Figure S7: Unbiased baseline trajectory for methylcarbene, showing the time evolution of the C–C–H angle, the spin-gap CV, and the switching parameter λ without metadynamics bias.

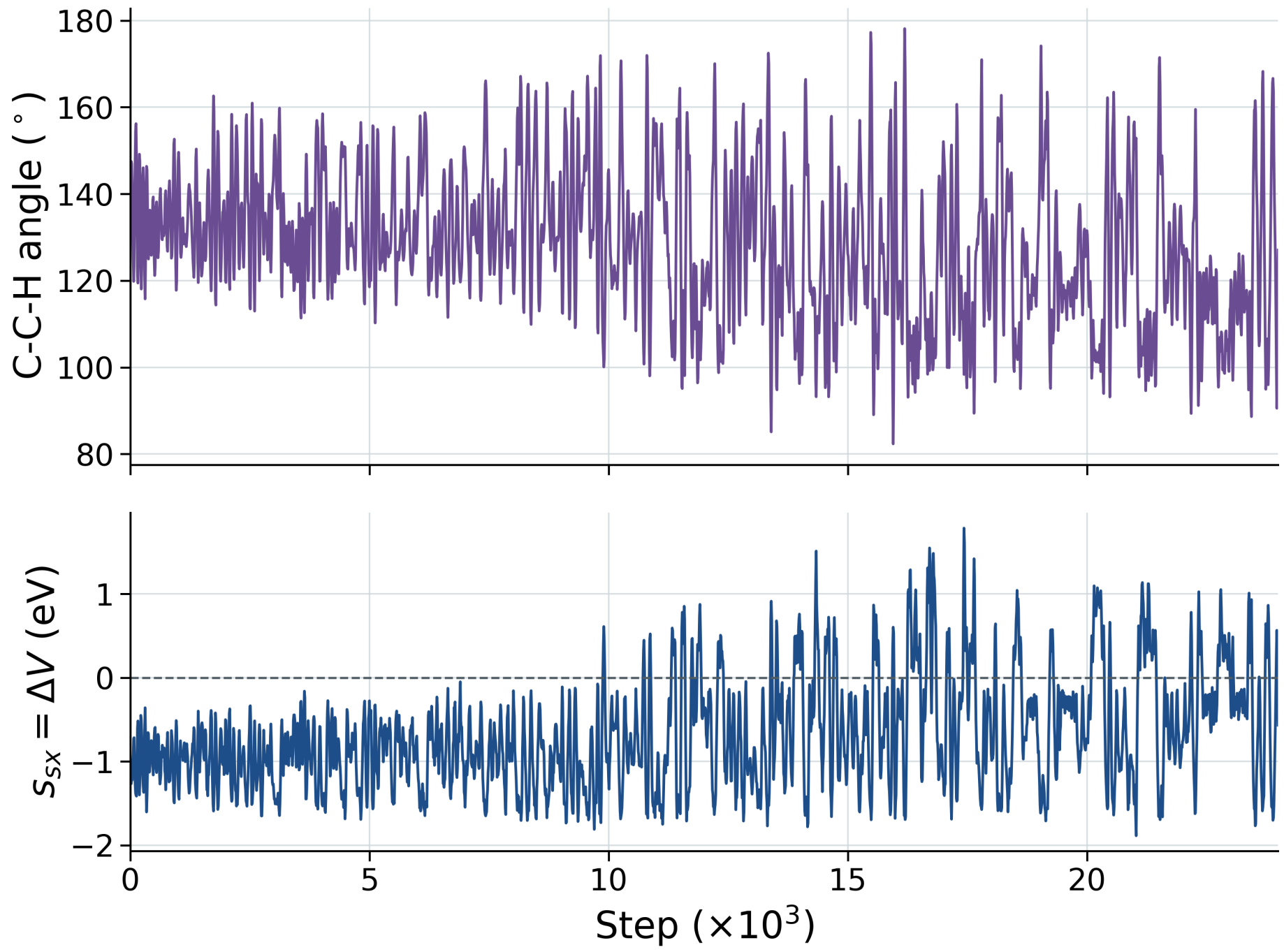

Figure S8: Evolution of the C–C–H angle along the methylcarbene DFT-based metadynamics trajectory, illustrating the close coupling between the structural angle and the spin-gap CV.

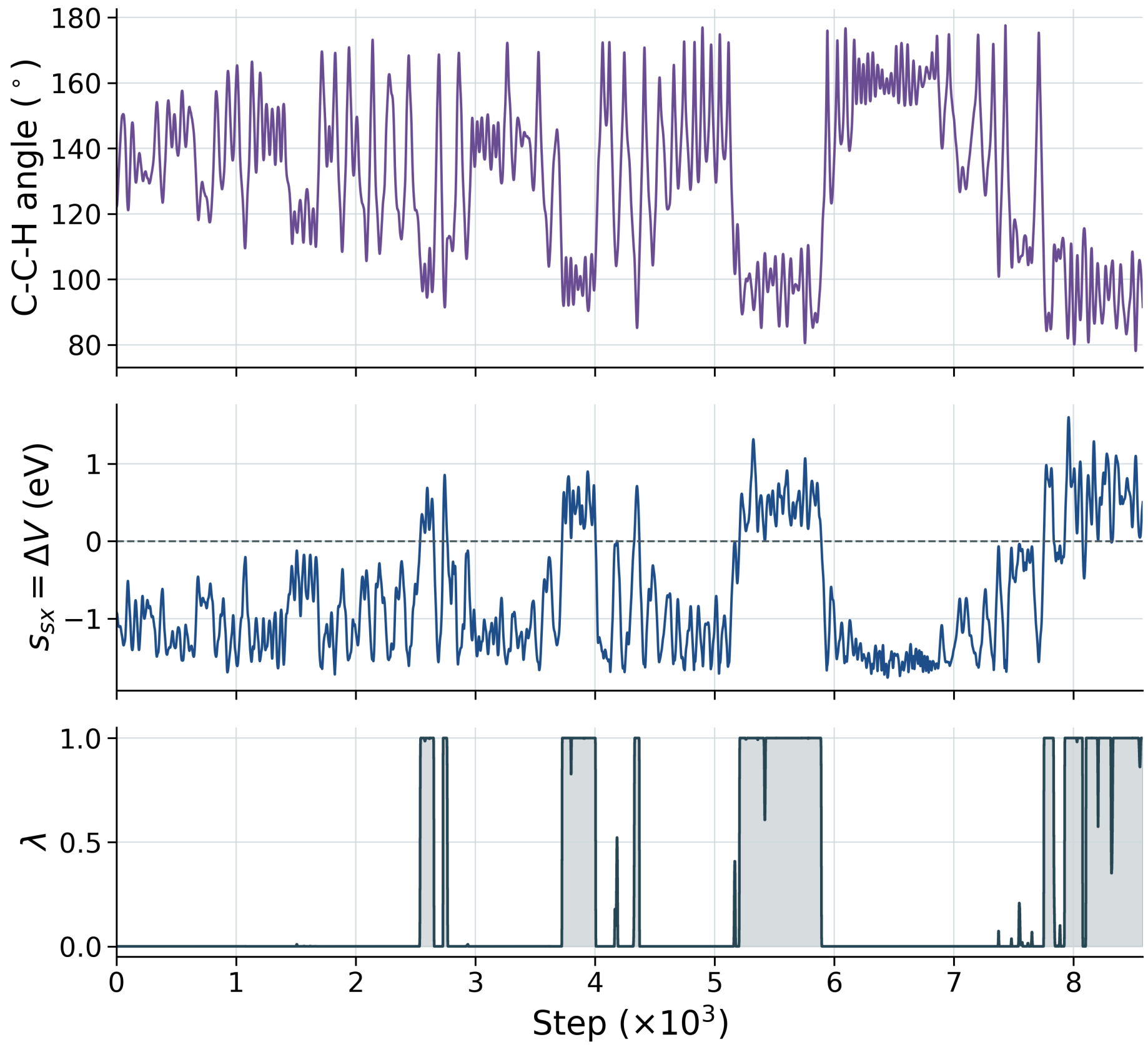

Figure S9: Evolution of the C–C–H angle, the spin-gap CV ($s_{\rm sx}$), and the switching parameter λ along a short DFT-based metadynamics trajectory of methylcarbene in which the C–C–H angle, instead of $s_{\rm sx}$, is used as the biased CV. The repeated changes in sign of $s_{\rm sx}$ and the corresponding changes in λ show that biasing this structural coordinate can drive the system across the spin-crossing region. The simulation parameters were consistent with those used in the other simulations, but the Gaussian width and height were set to 0.1 rad and 0.05 eV, respectively.

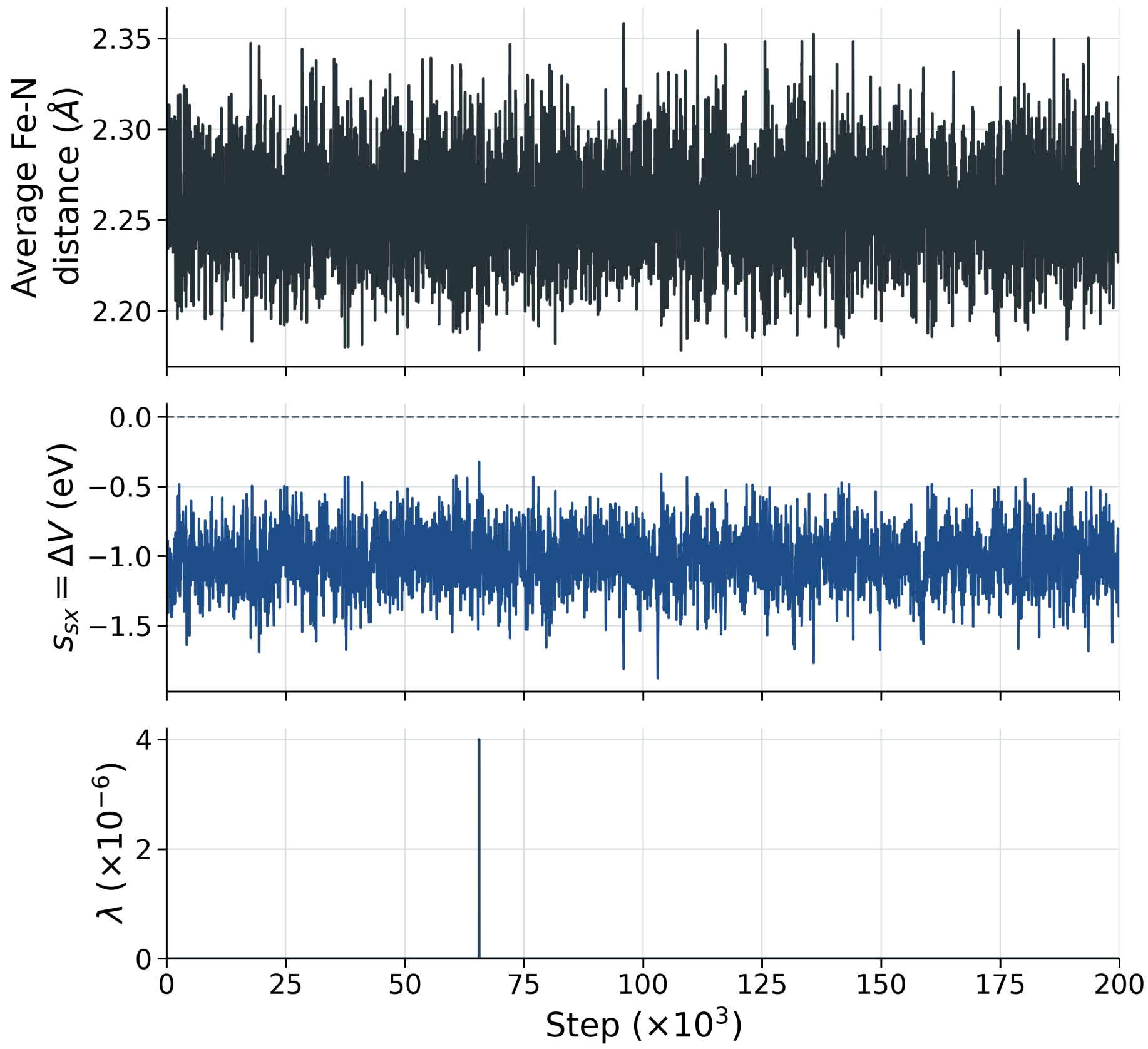

Figure S10: Unbiased baseline trajectory for $[Fe(2\text{-pic})_3]^{2+}$, showing the time evolution of the average Fe–N distance, the spin-gap CV, and the switching parameter λ without metadynamics bias.
